# Lenalidomide regulates CNS autoimmunity by promoting M2 macrophages polarization

**DOI:** 10.1038/s41419-018-0290-x

**Published:** 2018-02-14

**Authors:** Qinjie Weng, Jiaying Wang, Jiajia Wang, Jing Wang, Fahmida Sattar, Zhikang Zhang, Jiahuan Zheng, Zijie Xu, Mengting Zhao, Xuan Liu, Lijun Yang, Guifeng Hao, Liang Fang, Q. Richard Lu, Bo Yang, Qiaojun He

**Affiliations:** 1 Institute of Pharmacology & Toxicology, Zhejiang Province Key Laboratory of Anti-Cancer Drug Research, College of Pharmaceutical Sciences, Zhejiang University, Hangzhou, China; 2 Center for Drug Safety Evaluation and Research, Zhejiang University, Hangzhou, China; 3 Department of Rheumatology, Zhejiang Provincial People’s Hospital, Hangzhou, China; 4 Cancer Research Program, Max Delbrueck Center for Molecular Medicine in the Helmholtz Society, Berlin, Germany; 5 Department of Pediatrics, Brain Tumor Center, Cancer and Blood Disease Institute, Cincinnati Children’s Hospital Medical Center, Cincinnati, OH USA

## Abstract

Multiple sclerosis (MS) is a chronic and debilitating neurological disorder of the central nervous system (CNS), characterized by infiltration of leukocytes into CNS and subsequent demyelination. Emerging evidences have revealed the beneficial roles of M2 macrophages in ameliorating experimental autoimmune encephalomyelitis (EAE), a model for MS. Here, we identify that lenalidomide alone could promote macrophages M2 polarization to prevent the progression of EAE, which is associated with subsequent inhibition of proinflammatory Th1 and Th17 cells both in peripheral lymph system and CNS. Depletion of macrophages by pharmacology treatment of clodronate liposomes or transferring lenalidomide-induced BMDMs in EAE mice completely abolished the therapeutic effect of lenalidomide or prevented EAE development, respectively. The macrophages-derived IL10 was upregulated both in vivo and in vitro after lenalidomide treatment. Moreover, lenalidomide-treated IL10-dificient EAE mice had higher clinical scores and more severe CNS damage, and intravenous injection of lenalidomide-treated IL10^−/−^ BMDMs into mice with EAE at disease onset did not reverse disease severity, implying IL10 may be essential in lenalidomide-ameliorated EAE. Mechanistically, lenalidomide significantly increased expression and autocrine secretion of IL10, subsequently activated STAT3-mediated expression of Ym1. These studies facilitate the development of potential novel therapeutic application of lenalidomide for the treatment of MS.

## Introduction

Multiple sclerosis (MS) is an autoimmune inflammatory demyelinating disease, pathologically characterized by perivascular CD4^+^ T cells and monocytes inflammation, resulting in axonal demyelination and transection^1–3^. Current therapy of MS such as fingolimod, glatiramer acetate mainly depends on non-specific suppression of the immune system to delay the progression^4^ and reduce the frequency and severity of disease relapse periods. However, they cannot protect myelin from future damage^5,6^. Thus, more effective pharmacological strategies for MS to reduce inflammation and diminish myelin damage in injured CNS is urgently needed.

In recent years, it has become clear that macrophages play a very important role in the pathogenesis of MS^7^. The function of macrophages in experimental autoimmune encephalomyelitis (EAE), a widely used animal model of MS^8^, is often opposite^9^. M1 cells are classically activated macrophages, generally exhibit proinflammatory activity, whereas M2 cells are alternatively activated macrophages, predominantly inhibit immune responses^10^. M1 and M2 macrophages co-exist in vivo and present plastic properties regulated by members of the STAT family in the peripheral lymph system and CNS during the disease progress. STAT1/NF-κB axis promotes IFNs and Toll-like receptor (TLR) to skew macrophage function toward the M1 phenotype, whereas STAT3/STAT6 signaling pathway activated by IL4, IL10, or IL13 induces M2 macrophages phenotype^11^. The distribution of M1 macrophages in CNS lesion causes oligodendrocyte precursor cells death^12^ while M2 macrophages induce pro-repair molecules such as brain-derived neurotropic factor (BDNF), IL10, and ferritin increasing the maturation of oligodendrocyte precursor cells^13,14^. Moreover, M2 macrophages remove inhibitory debris or toxic products enabling subsequent remyelination^15^. Macrophages interactions with CD4^+^ T cells in demyelinating lesions have been suggested to play a critical role in modifying the pathobiology of MS^16^. As professional antigen-presenting cells (APCs), M1 macrophages prime naive CD4^+^ T cells and initiate auto-reactive immune response in CNS^17^, releasing many inflammatory mediators such as interleukin-1 (IL-1)^18^, tumor necrosis factor-alpha (TNF-α)^19^, and NO^20^, that are critical for EAE development^21^. However, M2 macrophages suppress CD4^+^ T cells activity in demyelinated CNS. Indeed, transferring of microglia- or monocyte-derived M2 macrophages could reduce the severity of clinical signs of EAE by inhibiting T cell proliferation^22^. The transition from M1 to M2 macrophages has been reported to be crucial for preserving myelin integrity^12^. Thus, the modulations of molecules associated with macrophage plasticity and polarized activation are critical to modulate CNS inflammation and tissue repair.

Here, we identified that lenalidomide, an oral drug approved by FDA for the treatment of myelodysplastic syndromes and multiple myeloma^23,24^, markedly promoted the M2 polarization of macrophages alone thus mediated immunosuppressive and neuroprotective effect in EAE. Moreover, lenalidomide induced M2 macrophages polarization mainly through expression and autocrine secretion of IL10 and subsequent activation of STAT3.

## Results

### Lenalidomide alone directly induces M2 phenotype in macrophages

**Fig. 1:**
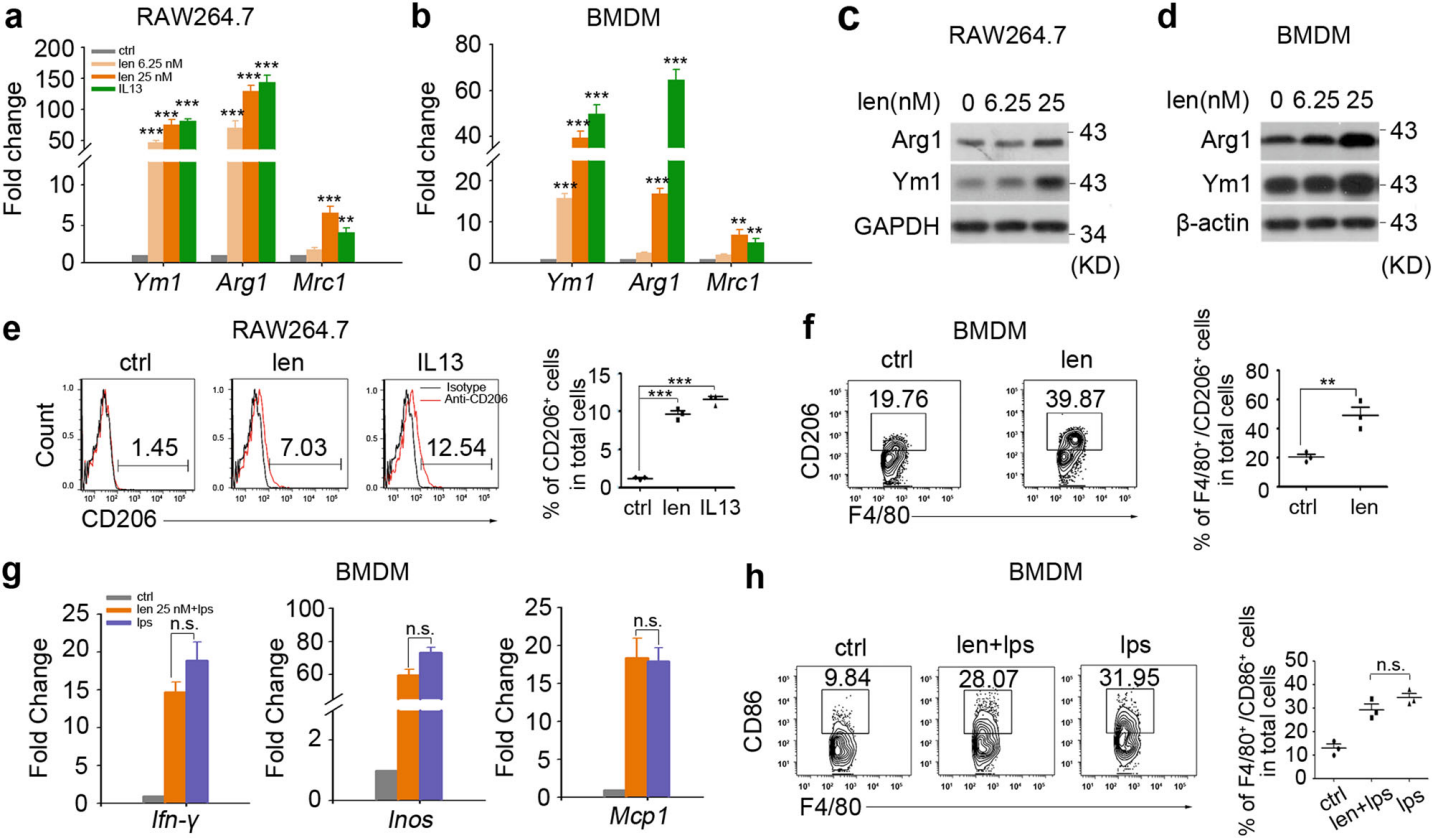
Lenalidomide promotes M2 macrophages polarization. **a**,** b** qRT-PCR analysis for expression of macrophage M2 phenotype genes in RAW264.7 (**a**) and BMDMs (**b**) with lenalidomide treatment for 4 h, IL13 (10 ng/ml) was used as positive control (*n* = 3). **c**, **d** Western blotting analysis for expression of Arg1 and Ym1 in RAW264.7 (**c**) and BMDMs (**d**) with lenalidomide treatment for 4 h. GAPDH or β-actin was the loading control (*n* = 3). **e** Flow cytometry analysis of the percentage of M2 phenotype (CD206^+^ cells) in RAW264.7 cells with lenalidomide (25 nM) treatment for 4 h. IL13 (10 ng/ml) was used as positive control (*n* = 3). **f** Flow cytometry analysis of the percentage of M2 phenotype (CD206^+^F4/80^+^ cells) in BMDMs with lenalidomide (25 nM) treatment for 4 h (*n* = 3). Cells were gated at F4/80^+^ cells. **g**, **h** BMDMs were pretreated with LPS (50 ng/ml) for 24 h and then administrated with lenalidomide (25 nM) for additional 4 h. qRT-PCR was carried out to analyze mRNA level of macrophage M1 phenotype genes (*n* = 3) (**g**). Flow cytometry analysis was carried out to characterize the percentage of M1 phenotype (CD86^+^F4/80^+^ cells), cells were gated at F4/80^+^ cells (*n* = 3) (**h**). Data are presented as means ± SEM; *P < 0.05, **P < 0.01, ***P < 0.001 versus untreated control

Recent studies have revealed that the balance between activation and polarization of M1 and M2 macrophages is important for the progression of EAE. Polarized macrophages can be modulated by pharmacological intervention. Therefore, we employed qRT-PCR experiment screening a variety of compounds/drugs, including anti-metabolites (pemetrexed and gemcitabine), anti-histamine drugs (loratadine and promethazine), 5-HT receptor antagonists (azasetron and palonosetron), immunomodulators (lenalidomide and pomalidomide), and novel compounds (referred to as C1–C2) in bone marrow-derived macrophages (BMDMs) in order to find a compound/drug, which selectively induces M2 phenotype or inhibits M1 phenotype. We found that the upregulation of *Arg1* and *Mrc1* mRNA level induced by lenalidomide, about 27.3 and 6.7 times, is closer to that induced by IL13, while pomalidomide upregulated the mRNA level of *Arg1* and *Mrc1* only by 18.6 and 3.9 times, respectively (Supplementary Figure S1a, b). Those compounds/drugs could not markedly inhibit the mRNA level of *Inos* and *Cxcl10* induced by LPS stimulation (Supplementary Figure S1c, d). The further qRT-PCR analysis demonstrated lenalidomide significantly increased the mRNA level of M2 macrophages-related genes of *Ym*1, Ar*g1* and *Mrc1* in RAW264.7 (monocyte-derived macrophage cell line), as well as in BMDMs (Fig. 1a, b and Supplementary Figure S2a). And western blotting analysis showed that lenalidomide enhanced the expression of Arg1 and Ym1 in macrophages (Fig. 1c, d). Moreover, flow cytometry analysis indicated that lenalidomide treatment significantly increased CD206^+^ M2 cells (Fig. 1e, f). Nevertheless, lenalidomide did not inhibit LPS-induced M1 polarization in BMDMs (Fig. 1g, h and Supplementary Figure S2b). Taken together, our results clearly demonstrate that lenalidomide alone could obviously increase M2 macrophages polarization.

### Lenalidomide ameliorates EAE symptom and reduces demyelination

**Fig. 2:**
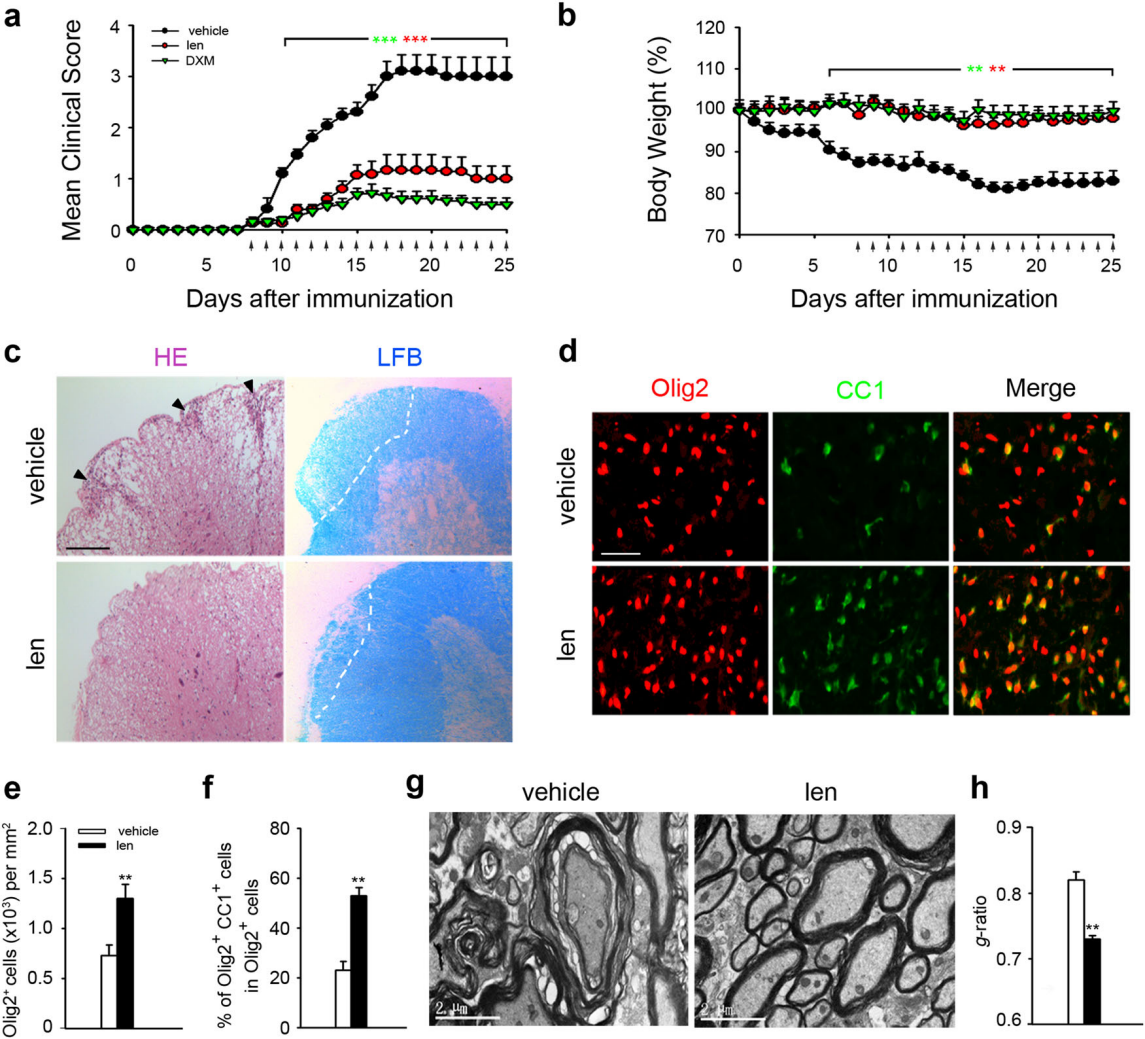
Lenalidomide ameliorates EAE progression. **a**, **b** Wild-type (WT) mice were immunized with MOG_35–55_ peptide and treated with lenalidomide (30 mg/kg, i.g.), dexamethasone (10 mg/kg, i.g.), or vehicle (0.9% CMC-Na, i.g.) at indicated time points (arrows). Mean clinical score (**a**) and loss of body weight (**b**) are shown (*n* = 15 per group). **c** H&E and Luxolfast blue (LFB) staining of mouse spinal cords from vehicle- and lenalidomide-treated WT EAE mice at day 16. Arrowheads indicate leukocyte infiltration area in H&E staining. Outlines indicate demyelination in LFB staining. Scale bars: 100 μm. **d** Immunostaining of mouse spinal cords from vehicle- and lenalidomide-treated WT EAE mice at day 16 using antibodies against Olig2 (red) and CC1 (green). Scale bars: 50 μm. **e** Quantification of Olig2^+^ cells density in spinal cords in **d**. **f** Quantification of the percentage of Olig2^+^CC1^+^ cells among Olig2^+^ cells from spinal cords in **d**. **g** Representative electron micrographs of spinal cords from vehicle- and lenalidomide-treated WT EAE mice at day 16. Scale bars: 2 μm. **h** g-ratios of axons in spinal cords from vehicle- and lenalidomide-treated WT EAE mice at day 16 (*n* > 100). Data are presented as means ± SEM; *P < 0.05, **P < 0.01, ***P < 0.001 versus vehicle-treated controls

Given that M2 macrophages have effect on inflammation resolution and tissue repair in EAE^25^, we tested whether lenalidomide might be able to ameliorate the development of autoimmune diseases. Therefore, C57BL/6 mice were immunized with MOG_35–55_ peptide and treated daily with lenalidomide after onset of clinical symptoms. Expectedly, lenalidomide ameliorated the disease severity from the early stage and lasted until the end of experiment, whereas vehicle-treated mice showed continued disease progression (Fig. 2a, b). Moreover, inflammatory foci areas in spinal cord of lenalidomide-treated mice were reduced compared with vehicle-treated mice (Fig. 2c, left). The inflammatory cells will attack the myelin to cause demyelination^26^, we next detect the myelin-forming cells and myelin integrity. Immunofluorescence analysis showed that the number of Olig2^+^ cells and Olig2^+^CC1^+^ cells in the spinal cords white matter of lenalidomide-treated mice compared with EAE control mice were significantly increased (Fig. 2d–f). Consistently, LFB analysis also indicated a decreased demyelination in the spinal cord of lenalidomide recipients in contrast to vehicle-treated EAE mice (Fig. 2c, right). Moreover, vehicle-treated EAE mice showed extensive demyelination compared to lenalidomide-treated EAE mice, with unraveling of the myelin sheaths and loss of axon (Fig. 2g). g-ratios (the ratio between the diameter of the axon to myelinated axon diameter) was used to quantify myelin thickness. Lenalidomide markedly decreased the g-ratios from 0.82 to 0.73 (Fig. 2h). These results indicate that lenalidomide could alleviate the symptoms of EAE mainly through decreased inflammatory cells infiltration in CNS and strong attenuating typical demyelination.

### Lenalidomide suppresses proinflammatory Th1 and Th17 cells responses

**Fig. 3:**
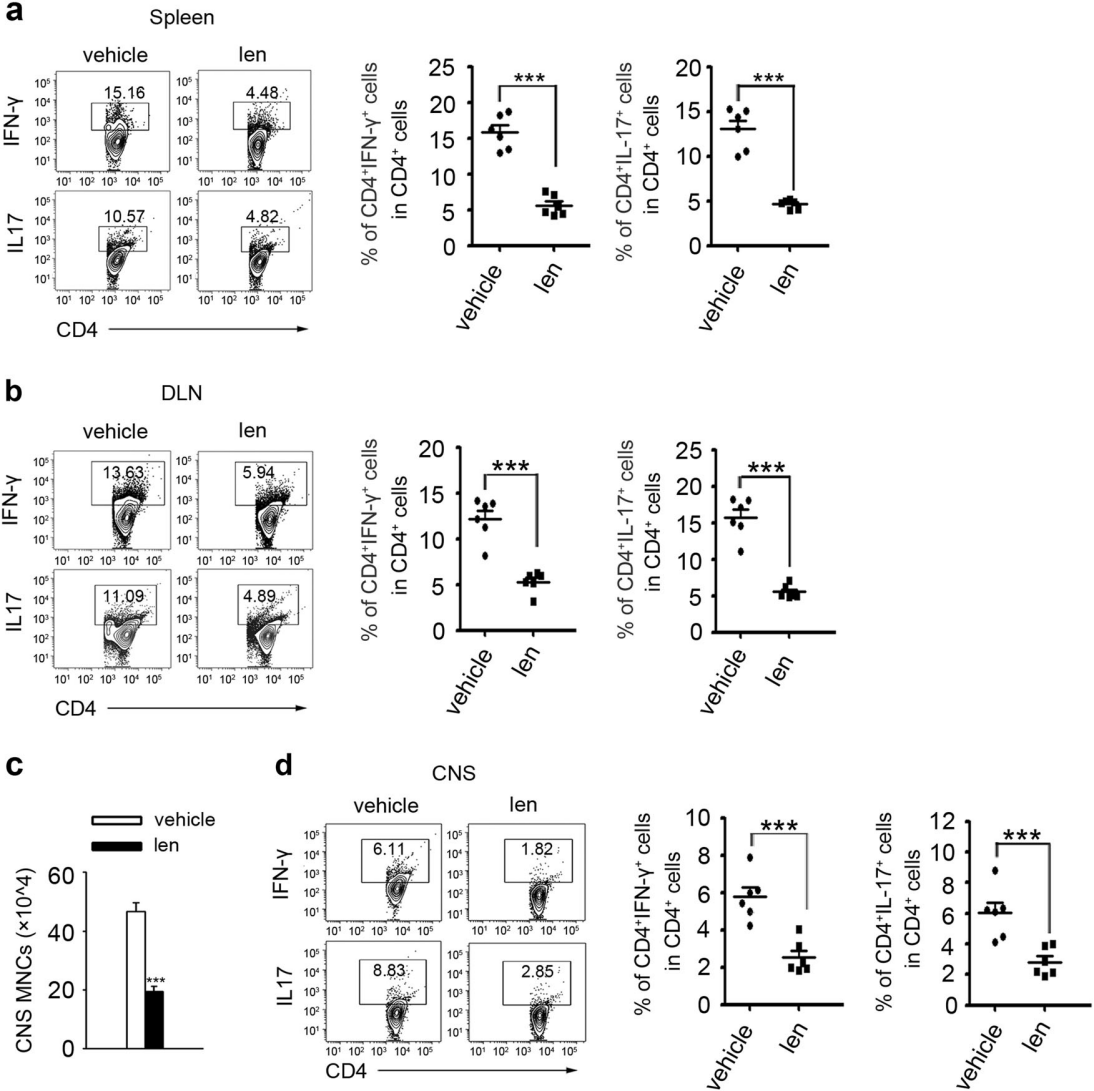
Lenalidomide suppresses autoimmune responses in EAE. **a**, **b** Flow cytometry analysis of Th1 and Th17 cells in spleen (**a**) and DLN (**b**) from vehicle- and lenalidomide-treated WT EAE mice at day 17. Representative fluorescence activated cell sorting (FACS) plots (left) and statistics from six mice per group (right) are shown; cells are gated for CD4^+^ T cells. **c** The total numbers of MNCs in whole spinal cord and brain were isolated from vehicle- and lenalidomide-treated mice on day 17 (*n* = 6). **d** Flow cytometry analysis of Th1 and Th17 cells in CNS-infiltrating MNCs from vehicle- and lenalidomide-treated WT EAE mice at day 17. Representative FACS plots (left) and statistics from six mice per group (right) are shown; cells are gated for CD4^+^ T cells. Data are presented as means ± SEM; *P < 0.05, **P < 0.01, ***P < 0.001 versus vehicle-treated controls

T cells and B cells contribute to autoimmune CNS inflammation and demyelination^27^, we therefore performed flow cytometry analysis to evaluated possible regulatory immune responses by lenalidomide in vivo. The experiments data demonstrated that lenalidomide treatment dramatically reduced IFN-γ^+^ and IL17^+^ CD4^+^ T cells in draining lymph node (DLN) and spleen (Fig. 3a, b), but had no effect on Foxp3^+^CD4^+^ T and B220^+^ B cells (Supplementary Figure S3a–d), suggesting that lenalidomide injection inhibited pathogenic Th1 and Th17 cells activity in vivo. To verify the modulated effect of lenalidomide on immune cells infiltration into CNS, we separated and quantified mononuclear cells (MNCs) from the whole spinal cord and brain at the peak phase of disease. In contrast to vehicle-treated EAE mice, the number of CNS-infiltrating MNCs in lenalidomide-treated EAE mice were reduced by 2.5 times (Fig. 3c). Similarly, flow cytometry analysis observed that Th1 and Th17 cells penetrated into CNS were threefold reduction in lenalidomide-injected mice (Fig. 3d). Taken together, we speculate that lenalidomide inhibits effector T cells polarization in spleen and DLN, which induces the reduction of inflammatory Th1 and Th17 cells penetrating into the CNS.

### Lenalidomide-attenuated EAE is dependent on polarized M2 macrophages

**Fig. 4:**
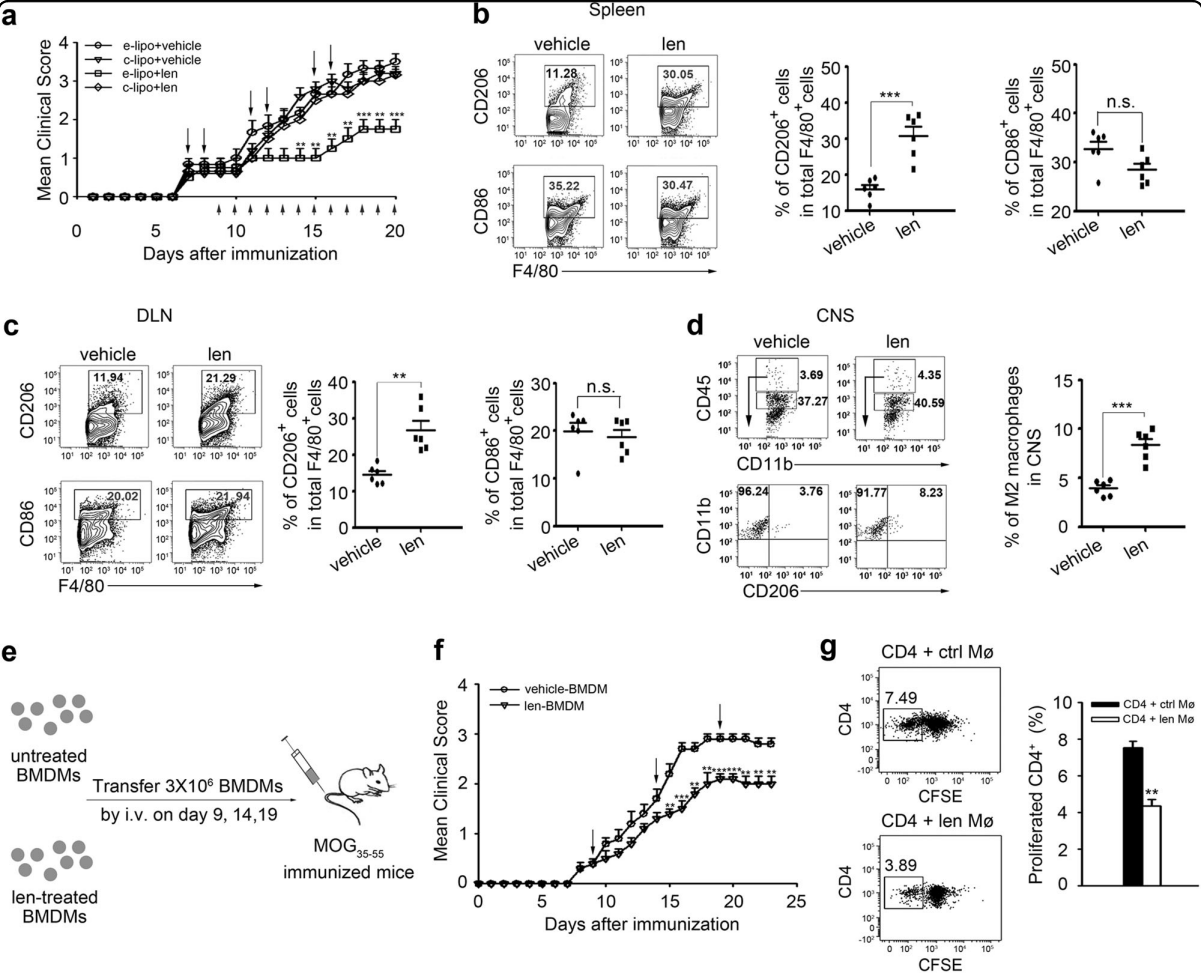
The protective effect of lenalidomide on EAE is dependent on M2 phenotype. **a** WT mice were immunized with MOG_35–55_ and treated with lenalidomide (30 mg/kg, i.g.) or vehicle (0.9% CMC-Na, i.g.) combined with empty or clodronate liposome (50 mg/kg, i.v.) at indicated time points (bottom arrows represent lenalidomide or vehicle treatment, upper arrows represent liposome treatment). Mean clinical score is shown (*n* = 15 per group). **b**, **c** Flow cytometry analysis of M2 and M1 macrophages in spleen (**b**) and DLN (**c**) from vehicle- and lenalidomide-treated WT EAE mice at day 17. Representative FACS plots (left) and statistics from six mice per group (right) are shown; cells are gated for F4/80^+^ cells. **d** Flow cytometry analysis of M2 macrophages in whole spinal cord and brain. CNS-infiltrating MNCs isolated from vehicle- and lenalidomide-treated WT EAE mice at day 17 stained for antibodies, including CD11b, CD206, and CD45. CD11b^+^CD45^hi^CD206^+^ cells were M2 macrophages. Representative FACS plots (left) and statistics from six mice per group (right) are shown. **e** WT mice were immunized with MOG_35–55_ on day 0 and 3 × 10^6^ BMDMs treated with or without 25 nM lenalidomide for 4 h were injected intravenously into these mice on day 9, 14, and 19 (*n* = 5 per group). **f** The mean clinical score of mice in **e**. **g** BMDMs were treated with or without 25 nM lenalidomide for 4 h. Splenic CD4^+^ T cells were isolated from MOG-treated WT mice, labeled with1 μM CFSE and subsequently cocultured with BMDMs at a ratio of 1:4 supplemented with MOG_35–55_ peptide (20 μg/ml) for 72 h. The proliferation of CD4^+^ T cells was confirmed by flow cytometry analysis based on CFSE dilution. Left panel represents flowcytometric dot plot of CFSE-labeled CD4^+^ T cells, right panel shows the percentage of CD4^+^ T cells that have proliferated based on CFSE dilution (*n* = 3 per group). Data are presented as means ± SEM; *P < 0.05, **P < 0.01, ***P < 0.001

To analyze whether macrophages related to the beneficial effect of lenalidomide in EAE, macrophages in spleen and spinal cord were selectively depleted using the “suicide technique” by application of clodronate liposomes (c-lipo). After administrating c-lipo, EAE mice could not be protected from disease progression upon lenalidomide treatment, particularly obvious at day 15 post immunization, clearly indicating that lenalidomide ameliorates CNS autoimmunity depending on the effect of macrophages (Fig. 4a). This hypothesis was further strengthened by the observation of macrophages subtypes in EAE mice. Reduction of disease severity in lenalidomide-treated mice was in paralleled with increased numbers of F4/80^+^CD206^+^ M2 cells both in spleen and DLN compared to vehicle-treated mice, however, the percentage of F4/80^+^CD86^+^ M1 cells remained unchanged (Fig. 4b, c). In addition, the frequency of activated CD11b^+^CD45^hi^CD206^+^ M2 macrophages in spinal cords was increased from 3.94 to 8.36% after treatment with lenalidomide (Fig. 4d). Based on our findings so far, we next transferred lenalidomide-pretreated BMDMs (Supplementary Figure S4a, b) into EAE mice every 5 days from the day of the appearance of clinical symptoms (Fig. 4e). After injection, adoptive transferred mice with M2 macrophages had lower clinical scores (Fig. 4f). The previous data showed that lenalidomide had effect on both CD4^+^ T cells and M2 macrophages in EAE mice, it is still unsure whether lenalidomide directly affects the function of myelin-specific CD4^+^ T cells or indirectly by regulating the function of macrophages. Indeed, we observed that CD4^+^ T cells were insensitivity to lenalidomide at a concentration of M2 cells polarization. However, higher concentration of lenalidomide (2 μM) augmented IFN-γ^+^CD4^+^ T cells (Supplementary Figure S5a, b), which is consistent with the previous studies^28^. Moreover, MOG-induced reaction of CD4^+^ T cells in vitro was not inhibited by lenalidomide (Supplementary Figure S5c), indicating that the therapeutic effect of lenalidomide on EAE mice was dependent on M2 macrophages polarization rather than direct inhibition of CD4^+^ T cells. Thus, we addressed whether lenalidomide-induced M2 macrophages have the capacity to inhibit myelin-specific CD4^+^ T cells, the result showed that MOG peptide-induced proliferation of CD4^+^ T cells was significantly inhibited when presented macrophages stimulated with lenalidomide (Fig. 4g). These results demonstrate that lenalidomide indirectly altering the proinflammatory activity of myelin-specific CD4^+^ T cells mainly through promoting the polarization of M2 macrophages.

### Macrophages-derived IL10 is essential for the therapeutic effect of lenalidomide on EAE

**Fig. 5:**
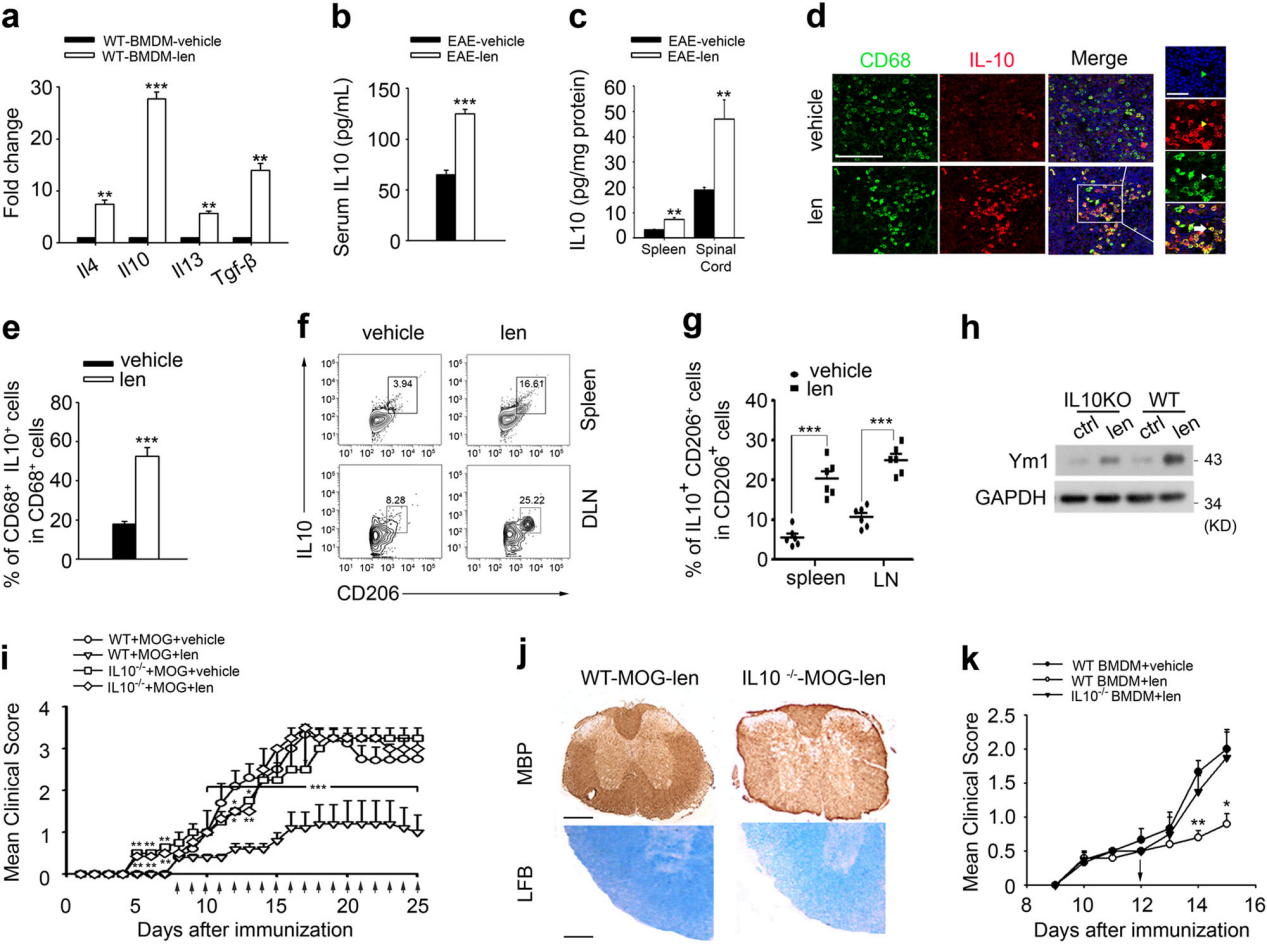
IL10-producing M2 macrophages play a critical role in lenalidomide treatment. **a** qRT-PCR analysis for expression of *Il4*, *Il10*, *Il13*, and *Tgf-β* in WT BMDMs with 25 nM lenalidomide treatment for 4 h (*n* = 3). **b** Concentration of IL10 in serum from vehicle or lenalidomide-treated WT EAE mice at day 17 (*n* = 6). **c** Concentration of IL10 in spleen and spinal cord from vehicle- or lenalidomide-treated WT EAE mice at day 17 (*n* = 6). **d** Immunostaining of mouse spinal cords from vehicle- and lenalidomide-treated WT EAE mice at day 16 using antibodies against CD68 (green) and IL10 (red). Scale bars: 150 μm (left), 50 μm (right). **e** Quantification of the percentage of CD68^+^ IL10^+^ cells among CD68^+^ cells from spinal cords in **d**. **f**, **g** Flow cytometry analysis of IL10^+^CD206^+^ cells in spleen and DLN from vehicle or lenalidomide-treated WT EAE mice at day 17. Representative FACS plots (**f**) and statistics from six mice per group (**g**) are shown. Cells are gated for CD206^+^ cells. **h** Western blotting analysis for expression of Ym1 in WT or IL10^−/−^ BMDMs with 25 nM lenalidomide treatment for 4 h. GAPDH was the loading control. **i** WT or IL10^−/−^ mice were immunized with MOG_35–55_ and treated with lenalidomide (30 mg/kg, i.g.) or vehicle (0.9% CMC-Na, i.g.) at indicated time points (arrows). Mean clinical score is shown (*n* = 15 per group). **j** MBP and LFB staining of spinal cord from lenalidomide-treated WT or IL10^−/−^ EAE mice in **i** at day 15. Scale bars: 200 μm (upper), 100 μm (bottom). **k** WT mice were immunized with MOG_35–55_ on day 0 and 3 × 10^6^ WT or IL10^−/−^ BMDMs treated with or without 25 nM lenalidomide for 4 h were injected intravenously into these mice at day 12. Mean clinical scores are shown (*n* = 8 per group). Data are presented as means ± SEM; *P < 0.05, **P < 0.01, ***P < 0.001

M2 macrophages secrete anti-inflammation cytokines including IL4, IL10, IL13, and TGF-β^10^. Based on this, we then tested whether lenalidomide was able to upregulate these factors. Expectedly, BMDMs stimulated with lenalidomide, confirmed by qRT-PCR analysis, led to a significant increase of *Il4*, *Il10*, *Il13*, and *Tgf-β* mRNA level, especially, *Il10 mRNA* increased about 25 times (Fig. 5a). Consistently, lenalidomide markedly increased IL10 production in serum, spleen, and spinal cord from EAE mice harvested at day 17 (Fig. 5b, c). IL10 can be produced in response to proinflammatory signals by virtually all immune cells, including T cells, B cells, and macrophages^29–31^. We conducted IHC and flow cytometry analysis to validate the source of IL10 in the presence of lenalidomide, the data revealed that the majority of IL10^+^ cells are also CD68^+^ macrophages (Fig. 5d–g), but not CD4^+^ T cells or CD8^+^ T cells (Supplementary Figure S6a–d), suggesting that IL10 was mainly produced by M2 macrophages. We next detected IL10’s role in lenalidomide-induced macrophages M2 polarization. In contrast to higher protein levels of Ym1 in IL10-sufficient BMDMs induced by lenalidomide, IL10-deficient BMDMs showed remarkably decreased Ym1 protein (Fig. 5h). To clearly distinguish whether the protective effect of lenalidomide was indeed mediated by IL10 in vivo, WT and IL10-deficient (IL10^−/−^) mice were immunized and treated with lenalidomide. Expectedly, IL10^−/−^ EAE mice treated with lenalidomide had higher mean clinical scores and more severe demyelination compared to lenalidomide-treated WT mice (Fig. 5i, j). Then, we used adoptive transfer experiment injecting lenalidomide-treated WT or IL10^−/−^ BMDMs to EAE mice on day 12. Consistent with in vivo data, IL10^−/−^ BMDMs were not able to inhibit the process of disease, while WT-BMDMs transferred mice displayed lower clinical scores (Fig. 5k). These results confirm that lenalidomide-ameliorated EAE is, at least partially, dependent on IL10-producing M2 macrophages.

### Lenalidomide promotes M2 macrophages polarization via IL10-STAT3-IL10 positive feedback loop

**Fig. 6:**
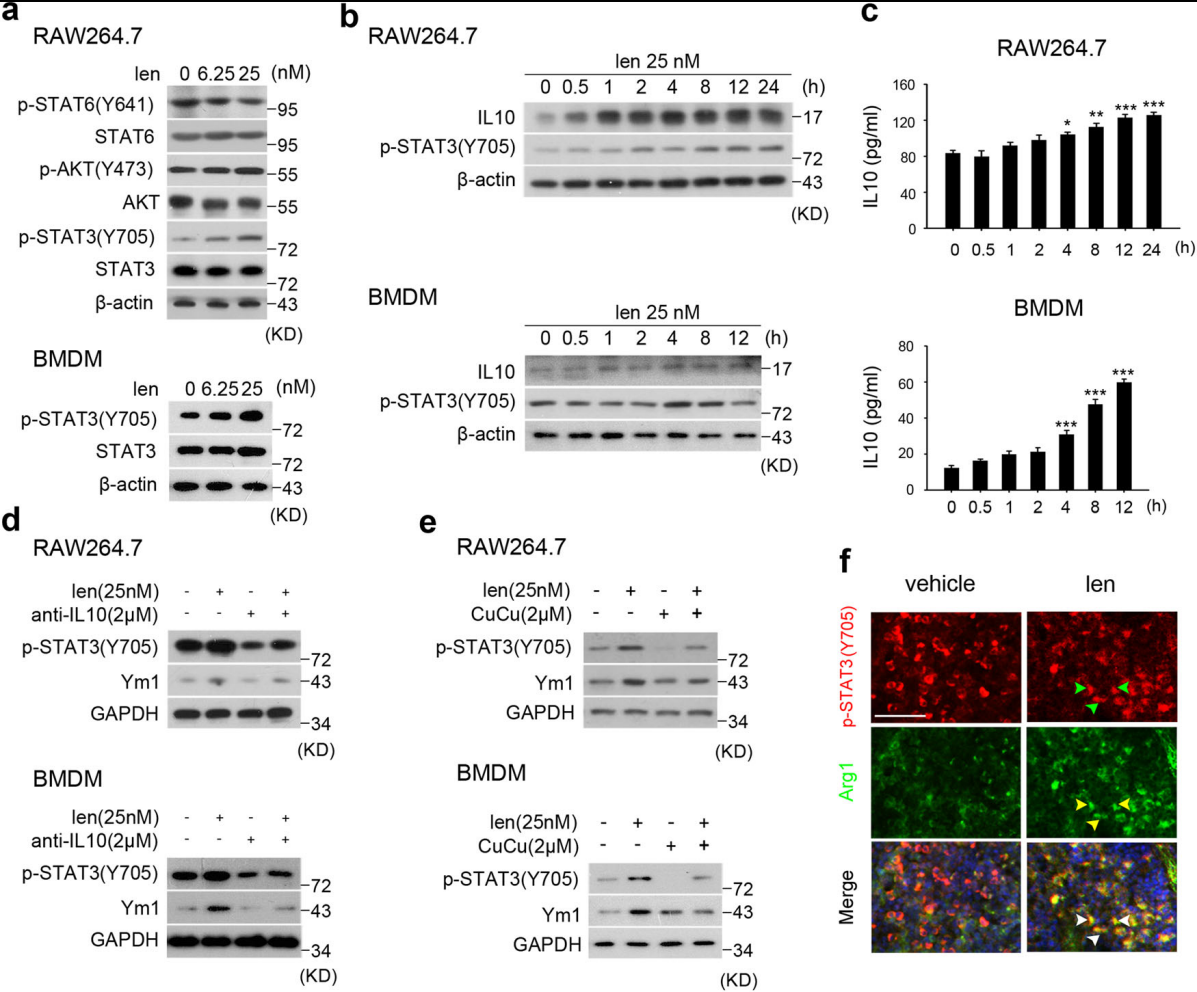
Lenalidomide-induced M2 macrophage polarization is dependent on STAT3. **a** Western blotting analysis for expression of STAT3, p-STAT3(Y705), AKT, p-AKT(Y473), STAT6, and p-STAT6 (Y641) in RAW264.7 (upper panel) and BMDMs (lower panel) treated with lenalidomide in different concentration for 4 h (*n* = 3). **b** Western blotting analysis for expression of IL10, p-STAT3 (Y705) in RAW264.7 (upper panel), and BMDMs (lower panel) with 25 nM lenalidomide treatment for a gradient time (*n* = 3). **c** RAW264.7 (upper panel) and BMDMs (lower panel) were cultured in serum-free medium and treated with lenalidomide for different times. Supernanant was cultured at indicated time to test the concentration of IL10 (*n* = 3). **d** RAW264.7 (upper panel) and BMDMs (lower panel) were cultured in serum-free medium, added anti-IL10 antibodies for 12 h and treated with lenalidomide for an additional 4 h. Western blotting analysis for expression of p-STAT3 (Y705) and Ym1. **e** RAW264.7 (upper panel) and BMDMs (lower panel) were pretreated with CuCu for 2 h and then treated with lenalidomide for an additional 4 h. Western blotting analysis for p-STAT3 (Y705) and Ym1. **f** Immunostaining of spleen from vehicle- and lenalidomide-treated EAE mice at day 16 using antibodies against p-STAT3 (Y705) (red) and Arg1 (green). Scale bars: 150 μm. GAPDH and β-actin were the loading control

Subsequently, we investigated how lenalidomide regulates M2 macrophages polarization. It has been reported that activated STAT6, as well as AKT and STAT3 can shift macrophages from the destructive M1 phenotype toward the beneficial M2 phenotype^11,32^. The western blotting analysis was performed in lenalidomide-treated RAW264.7 and BMDMs. The data showed that lenalidomide did not activate AKT and STAT6 in RAW264.7. However, lenalidomide upregulated STAT3 phosphorylation in a concentration-dependent fashion in both RAW264.7 cells and BMDMs (Fig. 6a). Moreover, IL10 protein level was upregulated then subsequently activated STAT3 in macrophages after lenalidomide treatment (Fig. 6b). We also detected that IL10 cytokine secretion began to increase after 4 h lenalidomide treatment (Fig. 6c). Moreover, pretreatment with recombinant anti-IL10 mAb suppressed Tyr-705 phosphorylation-mediated Ym1 expression. In addition, pretreatment with STAT3 inhibitor Cucurbitacin (CuCu) could partially reverse lenalidomide-stimulated macrophages M2 polarization (Fig. 6d, e). In vivo study confirmed that lenalidomide obviously increased Arg1^+^ p-STAT3^+^ cells in spleen of EAE mice (Fig. 6f). Accordingly, lenalidomide significantly increases expression and autocrine secretion of IL10, subsequently activates STAT3-mediated expression of Ym1.

## Discussion

**Fig. 7:**
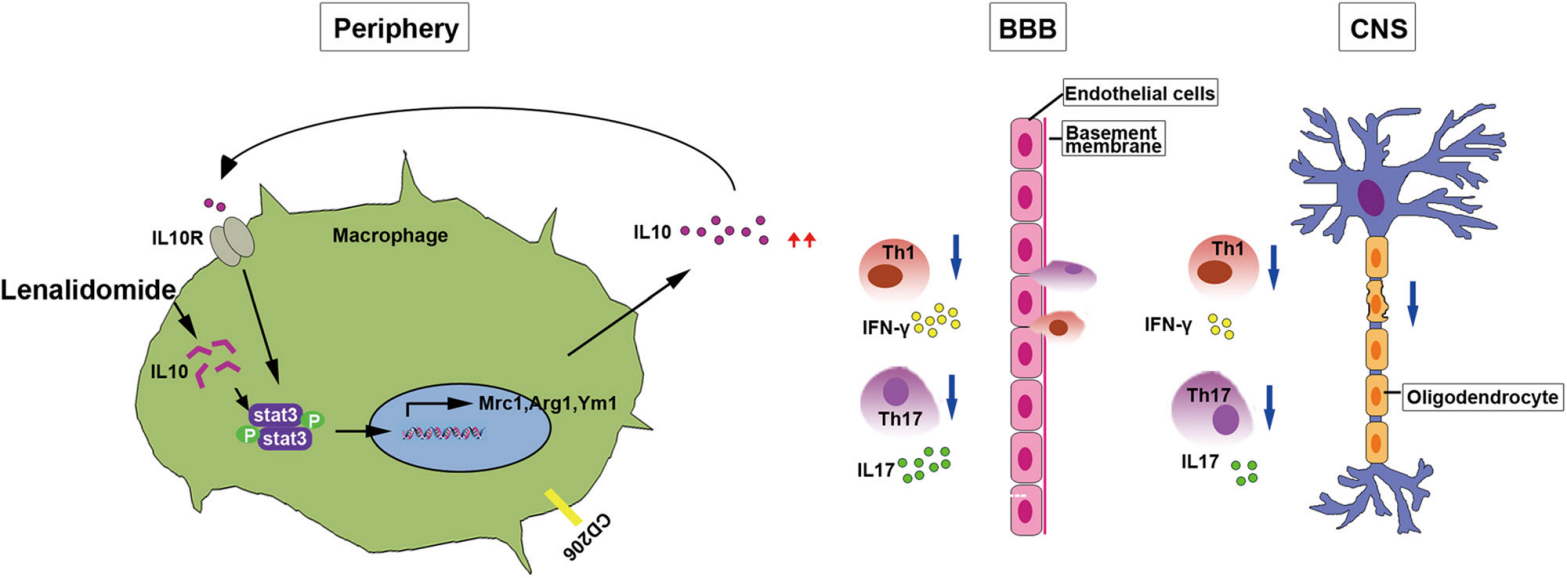
Schematic representation of lenalidomide-mediated M2 polarization and neuroprotective effect. Lenalidomide is uptaken by macrophages and directly promotes macrophages M2 polarization by IL10-STAT3 signaling pathway. Specifically, lenalidomide effectively increases IL10 expression and subsequently phosphorylates STAT3 Tyr-705. Phosphorylated STAT3 translocated into the nucleus encoding M2 macrophages-related genes such as Arg1, Ym1, and CD206. Anti-inflammatory cytokines secreted by M2 macrophages can reduce the infiltration of peripherally generated autoreactive Th1 and Th17 cells into CNS, inhibit demyelination, and therefore prevent of EAE

Here, we demonstrated that lenalidomide efficiently inhibited prototypical inflammatory demyelinating disease of CNS. Lenalidomide alone significantly activated M2 macrophages and inhibited proinflammatory Th1 and Th17 cells both in the peripheral lymph system and CNS, thus resulting in reduced inflammation and demyelination in injured CNS tissues. At the cellular level, lenalidomide efficiently promoted the expression and autocrine of IL10, subsequently phosphorylated STAT3 Tyr-705, and finally led to the induction of M2 macrophages (Fig. 7).

It has been reported that lenalidomide administration is neuroprotective in a mouse model of amyotrophic lateral sclerosis at symptom onset stage^33,34^, as well as in a transgenic model of Parkinson’s disease^35^ by decreasing the expression of the proinflammatory cytokines. Here, we found that administration of lenalidomide by gavage prevented EAE from the early stage and lasted until the end of experiment by reducing inflammation and demyelination. However, this observation was inconsistent with previous studies. The paper indicated that intraperitoneal administration of lenalidomide delayed symptom onset of EAE, but did not prevent demyelination^36^. We speculated that lenalidomide could not be fully dissolved in 1% carboxymethyl cellulose and 0.9% saline solution according to our formulation preparation (data not shown), lenalidomide by intraperitoneal administration was unable to fully absorbed in the abdominal cavity. Furthermore, the doses of lenalidomide was lower than we used in this paper.

Although many cytokines, including IL4, IL13, and IL10, efficiently direct M2 macrophages polarization^10^, they are not suitable for clinical use^37^. The previous trials that treated EAE mice with systemic IL10 did not show any promising effect and was found, at least in some studies, to aggravate disease^38^. A small quantity of compounds, such as pidotimod and azithromycin, could induce the polarization of M2 macrophages in an IL4- or IL13-depedent manner^39,40^; however, azithromycin had no therapeutic effect on EAE mice^41^. Here, we found that lenalidomide alone obviously induced M2 phenotype and did not affect M1 phenotype both in RAW264.7 and primary macrophages, however, whether lenalidomide modulates M2 macrophages polarization in human macrophages still need to be studied. Moreover, we observed that depletion of macrophages with c-lipo completely abolished the beneficial effects of lenalidomide on EAE while adoptive transferring of lenalidomide-induced macrophages ameliorated EAE, demonstrating that lenalidomide-alleviated inflammation and demyelination is mainly through promoting M2 macrophages polarization. Macrophages and CD4^+^ T cells both play important roles in the neuropathology of MS, and the interactions between CD4^+^ T helper cell subsets and M1 and M2 macrophages often result in particular functions and outcomes^16^. Autoreactive CD4^+^ T cells from peripherally infiltrate into the CNS, where they are restimulated by M1 macrophages, leading to local expansion of pathogenic Th1 and Th17 cells, and subsequently damage to oligodendrocytes^42^. Indeed, injection of M2 macrophages into mice with EAE has been shown to reduce the frequency and severity of disease relapse periods by suppressing T cell activity^22,43^. Exposing of T cells to lenalidomide leads to increased IFN-γ and IL-2 secretion^44^, however, we found that it did not directly inhibit MOG-loaded CD4^+^ T cells. Moreover, we here provided evidence that lenalidomide-induced M2 macrophages diminished the proliferation ability of MOG-specific CD4^+^ T cells ex vivo.

IL10 is an anti-inflammatory cytokine and its primary function is to limit inflammatory responses, which has been well studied in the pathogenesis of several autoimmune diseases including EAE^45^. Lenalidomide could reduce the survival support of NLCs for chronic lymphocytic leukemia (CLL) cells in vitro by increasing secretion of IL10^46^. Consistently, we showed here that lenalidomide promoted IL10 expression both in vivo and ex vivo in macrophages. IL10 played important roles in lenalidomide therapy as IL10^−/−^ mice could not be protected from actively induced EAE upon lenalidomide treatment. IL10 can be produced in response to proinflammatory signals by virtually all immune cells, including T cells, B cells, and macrophages^29–31^. Plasmablasts in the DLN serve as IL10 producers to limit autoimmune inflammation^47^ and CD4^+^ T cells-induced IL10 was associated with the beneficial effects of Bowman–Birk inhibitor (BBI) on EAE^48^. Here, we show that the effect of lenalidomide strictly depended on M2 macrophages-secreted IL10, as adoptive transferring of lenalidomide-activated IL10-sufficient macrophages improved EAE progress, however, IL10^−/−^ macrophages could not rescue EAE clinical scores. These findings emphasize the importance of M2 macrophages as IL10 producer for control of CNS autoimmunity. Up to now, the role of IL10 in macrophages has been clearly characterized and two important signaling axes have been reported to be activated through IL10 receptor ligation in macrophages, the JAK1/STAT3 and PI3K/Akt/GSK3β pathways^49,50^. We found here that lenalidomide-induced M2 macrophages depended on STAT3 activation. IL10/STAT3 axis is successfully studied in M2 macrophages, IL10 binding to IL10R^51,52^ activates the IL10/JAK1/STAT3 cascade, then phosphorylates STAT3 homodimers translocate to the nucleus within seconds to activate the expression of M2 macrophages-related genes^53^. STAT3 has pathological implications for MS due to its critical roles in myeloid cell activation, T cell polarization, and cytokine/chemokine induction^54,55^. However, whether hyperactivation of STAT3 in macrophages renders mice resistant to EAE disease need to be further studied.

Here, we found that therapeutic administration of lenalidomide significantly controls disease severity, and improves behavior in mice with EAE, suggesting that lenalidomide could be used for the treatment of inflammation-associated degenerative CNS diseases, such as relapsing-remitting MS. Lenalidomide is therefore a promising therapeutic drug candidate for attenuating inflammatory responses and reducing neuronal demyelination in CNS in MS.

## Materials and methods

### Animals and EAE mice induction

C57BL/6 mice, 6–8-week old, female, were purchased from Beijing Vital River Laboratory Animal Technology Co., Ltd. IL10^−/−^ mice, 6–8-week old, female, were purchased from Model Animal Research Center of Nanjing University. Those mice were immunized subcutaneously with 400 μg MOG_35–55_ peptide (Sangom Biotech) emulsified in CFA (Sigma) containing 4 mg/ml heat-killed Mycobacterium tuberculosis H37Ra (BD Biosciences) on day 0. In addition, 200 ng pertussis toxin (List Biological Laboratories) in 0.1 ml PBS was administered intravenously on day 0 and day 1 post immunization per mice. Animals were assessed daily for weight loss and clinical signs with a 0–5 point scoring system as follows: 0, healthy; 0.5, tip of tail is partial paralysis; 1, limp tail; 1.5, inhibition of hindlimb; 2, weakness of hindlimb; 2.5 dragging of hindlimb; 3, one hindlimb paralysis; 3.5, complete hindlimb paralysis; 4, one forelimb paralysis; 4.5, complete forelimb paralysis; and 5, severe paralysis or death.

### Cell culture

RAW264.7 cells were purchased from Cell Bank of Chinese Academy of Sciences, cultured in DMEM (GIBCO) containing 10% (vol/vol) FBS (GIBCO). BMDMs: bone marrow cells were isolated from femurs and tibias of C57BL/6 mice and cultured in DMEM containing 10% (vol/vol) FBS and 50 ng/ml of M-CSF (PEPROTECH) for 7 days. Purity was determined by flow cytometry.

### Drugs/compounds screening

Drug/compounds screening for promoting M2 macrophages polarization: BMDMs were seeded in 24-well plate presented with 100 nM drugs/compounds for 24 h. IL13 (10 ng/ml, PEPROTECH) was used as positive control for macrophages M2 polarization. Following qRT-PCR analysis for expression of *Arg1* and *Mrc1*. Drug/compounds screening for inhibiting macrophages M1 polarization: BMDMs were seeded in 24-well plate presented with LPS (50 ng/ml) for 24 h to induce M1 phenotype and then treated with drugs/compounds (100 nM) for additional 24 h. Following qRT-PCR analysis for expression of *Inos* and *Cxcl10*.

### Compounds and pharmacological treatment

For RAW264.7 and BMDMs, lenalidomide was dissolved in DMSO at proper concentration. For EAE mice, vehicle (0.9% CMC-Na, i.g.), dexamethasone (Tianjin Kingyork Group Co., Ltd, 10 mg/kg, i.p.) and lenalidomide (Chia Tai Tianqing Pharmaceutical Group Co., Ltd, 30 mg/kg in 0.9% CMC-Na, i.g.) were daily administrated after the appearance of clinical symptom (average clinical score ≥0.5). To selectively deplete macrophages, clodronate liposome (Encapsula, 50 mg/kg, i.v.) was administrated on day 7, 8, 11, 12, 15, and 16 after immunization.

### Suppression assays

BMDMs were generated as described above and on day 7 of differentiation, cells were treated with or without 25 nM lenalidomide. After 4 h, splenic CD4^+^ T cells were isolated from MOG-treated WT mice via magnetic separation (EasySep Mouse CD4 Isolation Kit, Stem Cell Technologies), labeled with1 μM CFSE (Biolegend) and subsequently cocultured with BMDMs at a ratio of 1:4 in RPMI 1640 (GIBCO) media supplemented with MOG_35–55_ peptide (20 μg/ml) for 72 h. The proliferation of CD4^+^ T cells was assessed by flow cytometry based on CFSE dilution.

### Adoptive transfer of macrophages

BMDMs from WT or IL10^−/−^ mice were cultured for 7 days and then treated with DMSO or lenalidomide for 4 h. Cells were collected and resuspended in DMEM without serum. In total, 3 × 10^6^ BMDMs were injected intravenously into EAE mice at specific days depending on the severity of disease.

### Immunohistology

Spinal cords and spleen from lenalidomide- or vehicle-treated EAE mice were embedded in OCT (SAKURA Finetek) and spinal cords were cut into 12 μm sections while spleen were cut into 8 μm sections. Immunofluorescence staining was performed and incubated with the appropriate dilutions of primary antibodies against Olig2 (Millipore), CC1 (Oncogene Research), CD68, CD4, CD8 (all purchased from Santa Cruz), IL10 (Abcam), Arg1, p-STAT3(705) (all purchased from Cell Signaling Technology). Subsequently, slides were incubated with Alexa Fluor (AF) 568- or AF488-coupled secondary antibodies (Life Technologies). Nuclei were counterstained with DAPI (Dojindo). For LFB staining, slides were stained with LFB solution (Sigma-Aldrich) overnight, rinsed with distilled water, and differentiated with 0.05% lithium carbonate solution followed by 70% ethanol. Differentiation was stopped by rinsing in distilled water. For HE staining, spinal cords from lenalidomide- or vehicle-treated EAE mice were embedded in paraffin and cut into 3 μm sections, deparaffinized and stained with hemotaxylin and eosin. For MBP IHC, slides were incubated with the appropriate dilutions of primary antibodies against MBP (Covance) and incubated with secondary antibody (Beijing Zhongshan Biotechnology) for 1 h before subjected to DAB staining. Images were taken by Zeiss LSM510 meta fluorescence confocal microscope or Leica DM2500 microscope.

### Electron microscope

Spinal cords from lenalidomide- or vehicle-treated EAE mice were fixed with 2.5% glutaraldehyde for 3 days. The tissues were then PBS washed, fixed in 1% osmium tetroxide for 1 h, and stained with 2% uranyl acetate for 30 min, subsequently dehydrated in graded ethanol series, and embedded in Epon. g-ratio defined as the ratio of the diameter of a given axon and the myelinated fiber diameter. Axon and axon plus myelin units were measured for more than 100 times for each group using ImageJ.

### Western blotting and ELISA

Protein extracts were subjected to SDS-PAGE (8–12% gels) and blotted onto PVDF membranes. After blocking with 5% fat-free milk, the membranes were incubated with the following antibodies: anti-AKT, anti-p-AKT, anti-Arg-1, anti-GAPDH, anti-β-actin (all purchased from Santa Cruz Biotechnology), anti-STAT6, anti-p-STAT6, anti-STAT3, anti-p-STAT3(705) (all purchased from Cell Signaling Technology), anti-Ym1, anti-IL10 (all purchased from Abcam) at 4 °C overnight. The bound antibodies were detected using horseradish peroxidase (HRP)-conjugated IgG (MULTI Sciences) and visualized with enhanced chemiluminescence (ECL, PerkinElmer) detection reagents (Thermo scientific, USA). β-actin or GAPDH was used as a loading control. IL-10 concentration in plasma, tissues, and cell culture supernatants were determined by ELISA kit (DAKEWE). The tissue ELISA measurements were normalized to the protein content of the homogenates.

### RNA isolation and qRT-PCR

Total RNA was isolated from cells with Trizol reagent (Invitrogen), cDNA was transcribed using TransScript kit (TransGen Biotech). qRT-PCR analysis was performed using the SYBR Green (Bio-Rad) method on the ABI Fast 7500 real-time PCR instrument (Perkin-Elmer Applied Biosystems). The gene expression was normalized to the expression of the gene encoding GAPDH.

### Monocytes isolation and flow cytometry analysis

Single-cell suspensions from spleen and draining lymph nodes (DLN) were obtained by mechanical disruption. For spleen cells, using NH_4_Cl lysis buffer to remove red blood cells. CNS-infiltrating monocytes were isolated from spinal cord and brains as follows. CNS tissues were cut into small pieces and digested in Hank’s balanced salt solution (HBSS) containing 20 mM Hepes (Sigma), 0.25% collagenase D (GIBCO), 0.025 U/ml DNaseI (Sigma) at 37 °C for 35 min with brief vortex mixing every 15 min. At the end of digestion, the solution was mixed thoroughly and the cells were washed twice in PBS and suspended in 30% Percoll, overlaid on 70% Percoll and centrifuged for 40 min at 1300 × *g* at 25 °C. Cells at the interface were CNS monocytes.

Single-cell suspensions from DLN, spleen, and CNS were incubated for 30 min at 4 °C with fluorochrome-conjugated anti-CD4, anti-CD8, anti-CD45, anti-B220 (all purchased from BD Biosciences), anti-F4/80, anti-CD11b, anti-CD86, and anti-CD206 (all purchased from Biolegend) for staining of surface markers. For intracellular staining of cytokines, the cells were stained with anti-CD4, followed by staining with anti-IFN-γ, anti-IL17A (all purchased from eBioScience) and anti-IL10 (Biolegend) using Cytofix/Cytoperm kit (BD Biosciences) according to the manufacturer’s protocol. Intracellular staining with anti-Foxp3 (eBioscience) was performed using an Fixation/Permeabilization kit (eBioscience) according to the manufacturer’s protocol.

### Data and statistical analysis

All data from at least three different experiments. All values were presented as means ± SEM, and statistically significant differences were assessed by one-way ANOVA. A value of *P* < 0.05 was considered statistically significant.

## Electronic supplementary material

supplementary material
