# Correction: Lenalidomide regulates CNS autoimmunity by promoting M2 macrophages polarization

**DOI:** 10.1038/s41419-020-2287-5

**Published:** 2020-02-07

**Authors:** Qinjie Weng, Jiaying Wang, Jiajia Wang, Jing Wang, Fahmida Sattar, Zhikang Zhang, Jiahuan Zheng, Zijie Xu, Mengting Zhao, Xuan Liu, Lijun Yang, Guifeng Hao, Liang Fang, Q. Richard Lu, Bo Yang, Qiaojun He

**Affiliations:** 1 Institute of Pharmacology & Toxicology, Zhejiang Province Key Laboratory of Anti-Cancer Drug Research, College of Pharmaceutical Sciences, Zhejiang University, Hangzhou, China; 2 Center for Drug Safety Evaluation and Research, Zhejiang University, Hangzhou, China; 3 Department of Rheumatology, Zhejiang Provincial People’s Hospital, Hangzhou, China; 4 Cancer Research Program, Max Delbrueck Center for Molecular Medicine in the Helmholtz Society, Berlin, Germany; 5 Department of Pediatrics, Brain Tumor Center, Cancer and Blood Disease Institute, Cincinnati Children’s Hospital Medical Center, Cincinnati, OH USA

**Correction to: Cell Death and Disease**

10.1038/s41419-018-0290-x published online 14 February 2018

Since online publication of this article, the authors noticed that there were errors in the images used to compile Figs. 1, 3 and Supplementary Figure S3. Specifically, incorrect western blot cropping resulted in the wrong images being used to compile the GAPDH bands in Fig. 1c, and the Ym1 and GAPDH bands in Fig. 1d. In addition, incorrect images were used to compile the flow cytometry images in Fig. 3d (CD4+IL-17+/lenalidomide) and Supplementary Figure S3d (CD4+Foxp3+/lenalidomide), resulting in erroneous duplication. The corrected figures are provided below. The authors apologise for any inconvenience caused, and have confirmed that the associated quantitation is correct and that the results of the study were not affected.

**Figure:**
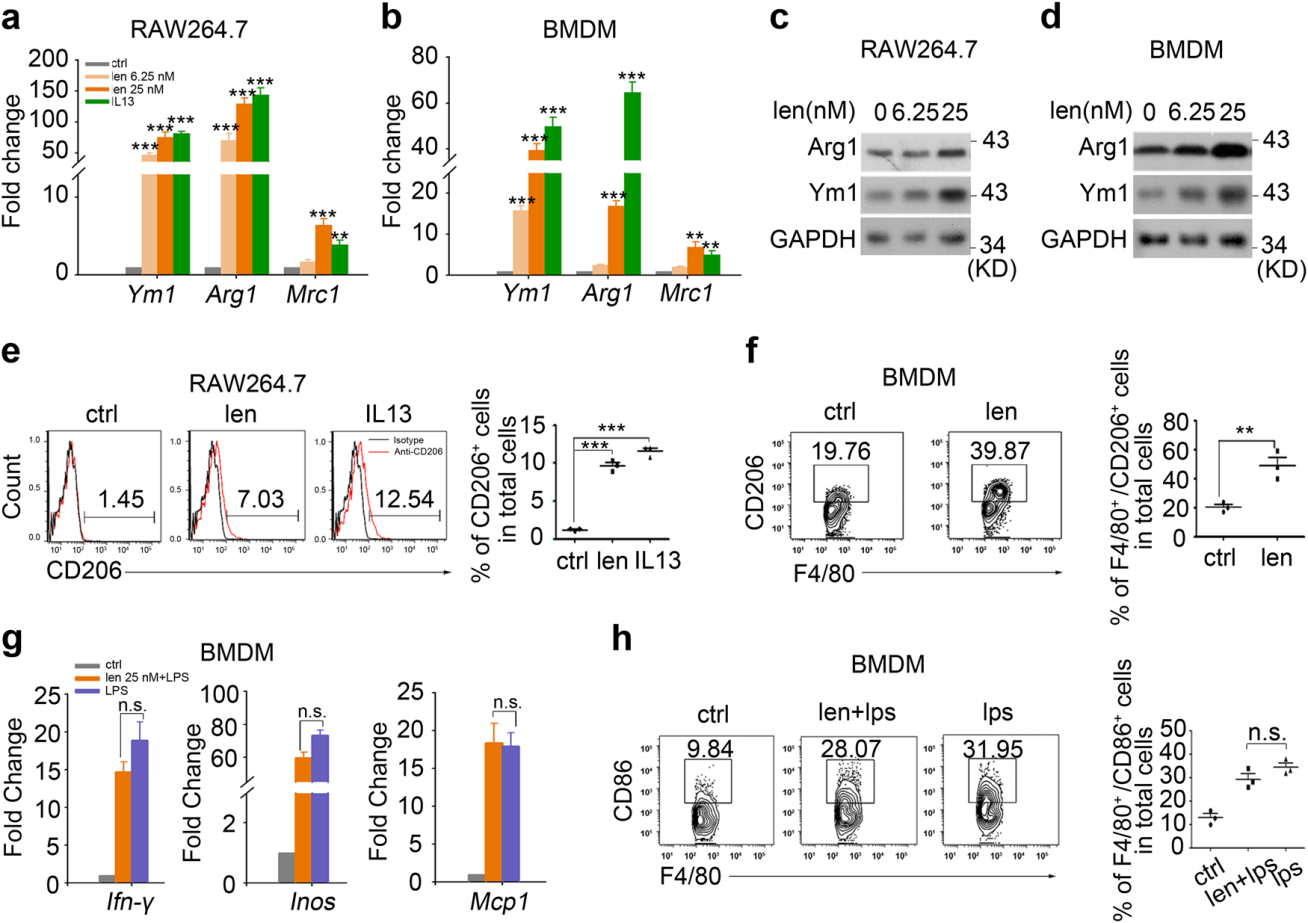
Fig. 1

**Figure:**
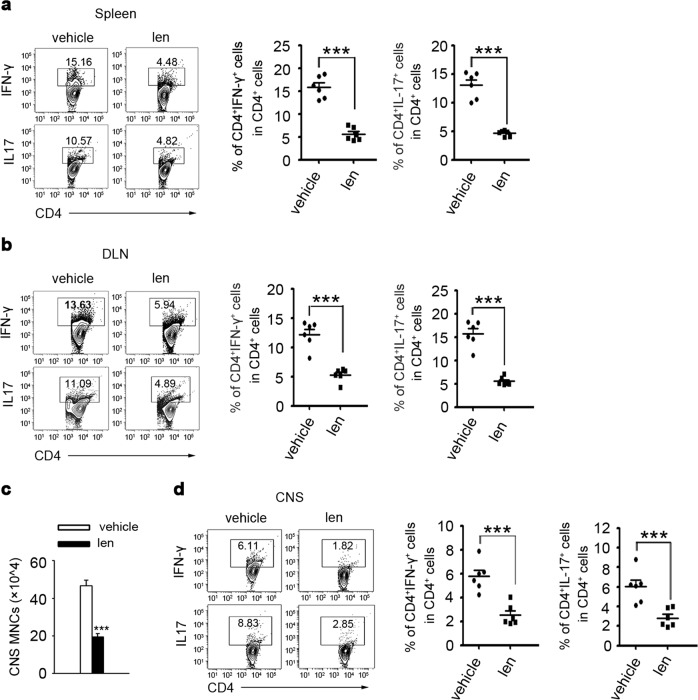
Fig. 3

**Figure:**
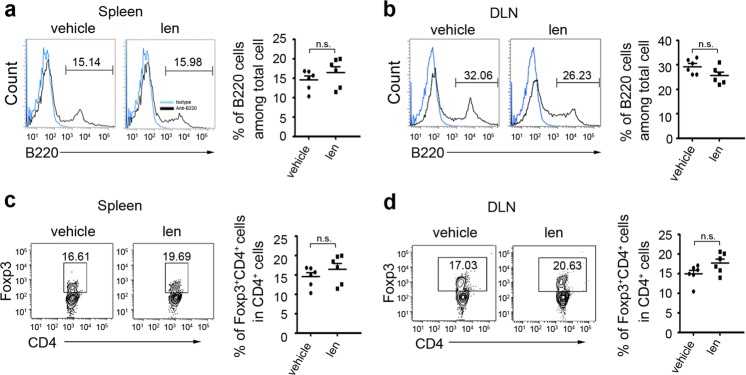
Fig. S3

