# Supplementary material for: Lenalidomide regulates CNS autoimmunity by promoting M2 macrophages polarization
Source: Cell Death Dis. 2018 Feb 14;9(2):251. doi: 10.1038/s41419-018-0290-x (PMC5833426; doi:10.1038/s41419-018-0290-x)
Supplement: Supplementary file 1 — supplementary material [file 41419_2018_290_MOESM1_ESM.docx]

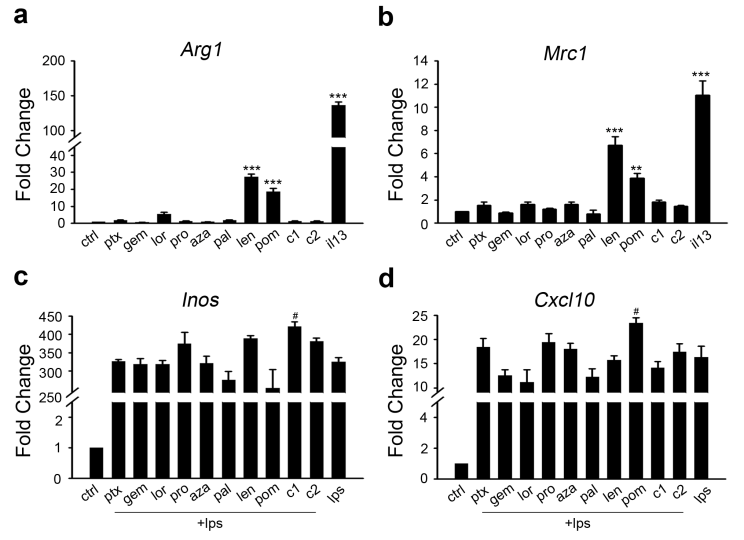


**Supplementary Figure 1** Drugs/Compounds screening that influence macrophages polarization. (**a** and **b**) BMDMs were treated with different drugs/compounds (100 nM) for 24h, qRT-PCR was carried out to analyze mRNA level of *Arg1* (**a**) and *Mrc1* (**b**). IL13 (10 ng/ml) was used as positive control. (**c** and **d**) BMDMs were pretreated with LPS (50 ng/ml) for 24h and then administrated with different drugs/compounds (100 nM) for additional 24h. qRT-PCR was carried out to analyze mRNA level of *Inos* (**c**) and *Cxcl10* (**d**)*.* LPS was used as positive control. Data are presented as means±S.E.M.; ***P*<0.01, ****P*<0.001 versus untreated control, ^#^*P*<0.05 versus LPS positive control.

­­­­­


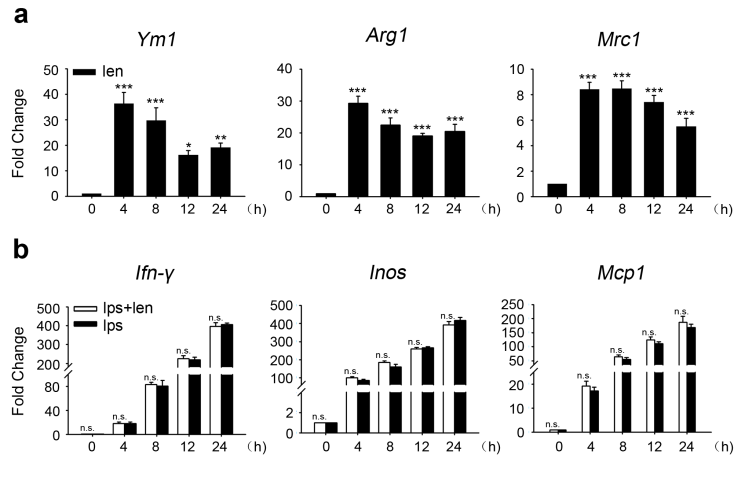


**Supplementary Figure 2** The effect of Lenalidomide on macrophages polarization. (**a**) BMDMs were treated with Lenalidomide (100 nM) for 4, 8, 12, 24h, qRT-PCR was carried out to analyze mRNA level of M2 phenotype genes*.* (**b**) BMDMs were pretreated with LPS (50 ng/ml) for 24h and then administrated with Lenalidomide (100 nM) for additional 4, 8, 12, 24h. qRT-PCR was carried out to analyze mRNA level of M1 phenotype genes*.* Data are presented as means±S.E.M.; **P*<0.05, ***P*<0.01, ****P*<0.001 versus untreated group­­­­­.


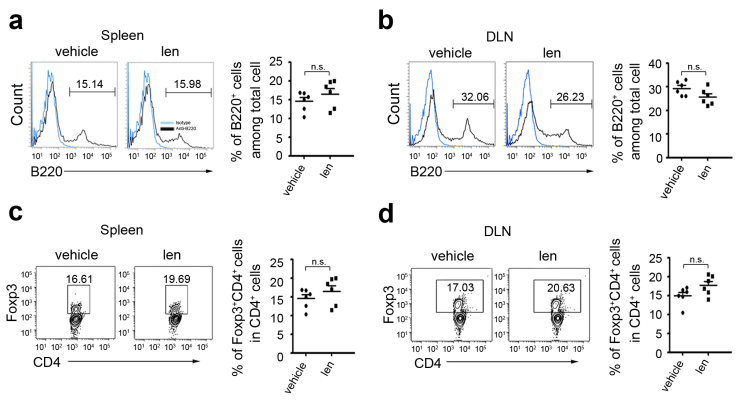


**Supplementary Figure 3** Lenalidomide treatment does not influence B cells and Treg cells in spleen and DLN in EAE. (**a** and **b**) Flow cytometry analysis of B220^+^ B cells in spleen (**a**) and draining lymph nodes (DLN) (**b**) from vehicle- and Lenalidomide-treated WT EAE mice at day 17 (*n*=6). Representative FACS (left) and statistics from 6 mice per group (right) are shown; cells are gated in total splenocytes and lymphocytes respectively. (**c** and **d**) Flow cytometry analysis of Treg cells in spleen (**c**) and DLN (**d**) from vehicle- and Lenalidomide-treated WT EAE mice at day 17(*n*=6). Representative FACS plots (left) and statistics from 6 mice per group (right) are shown; cells are gated for CD4^+^ T cells. Data are presented as means±S.E.M.


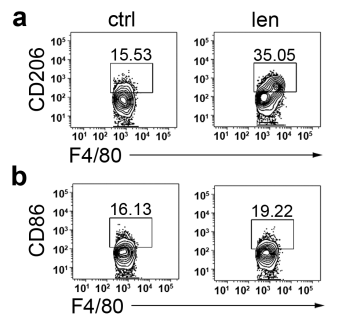


**Supplementary Figure 4** The ratio of M1 and M2 cells under Lenalidomide treatment. (**a** and **b**) Flow cytometry analysis of the percentage of M2 phenotype (CD206^+^F4/80^+^ cells) (**a**) and M1 phenotype (CD86^+^F4/80^+^ cells) (**b**) in BMDMs with vehicle or Lenalidomide (25 nM) treatment for 4h. Cells were gated at F4/80^+^ cells.


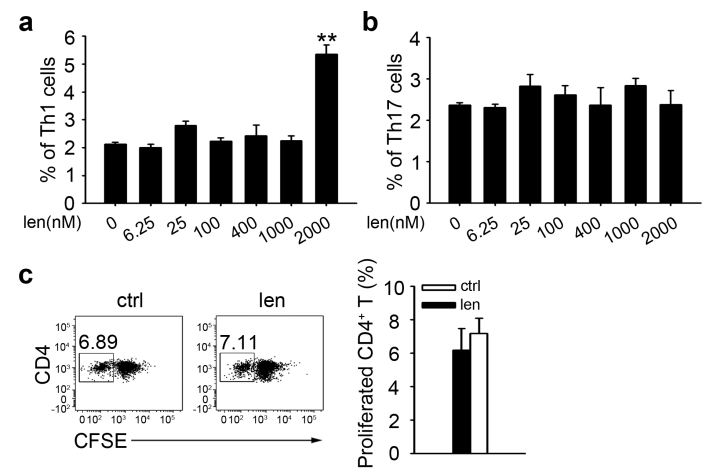


**Supplementary Figure5** Lenalidomide does not influence the proliferation of myelin-specific CD4^+^ T cells. (**a** and **b**) Splenic CD4^+^ T cells were derived from WT mice and treated with anti-CD3 (1 ug/ml) combined with vehicle or Lenalidomide for 72 h. The percentage of Th1 (**a**) and Th17 (**b**) cells were confirmed by flow cytometry analysis (*n*=3). (**c**) Splenic CD4^+^ T cells were isolated from WT EAE mice. To assess proliferation, CD4^+^ T cells were labeled with CFSE and stimulated with MOG_35–55_ peptide (20 μg/ml) combined with vehicle or Lenalidomide for 72 h. CFSE dilution was assessed by flow cytometry. Left panel represents flowcytometric dot plot of CFSE labeled CD4^+^ Tcells, right panel shows the percentage of CD4^+^ T cells that have proliferated based on CFSE dilution (*n*=3). Data are presented as means±S.E.M.; ***P*<0.01 versus untreated group­­­­­.


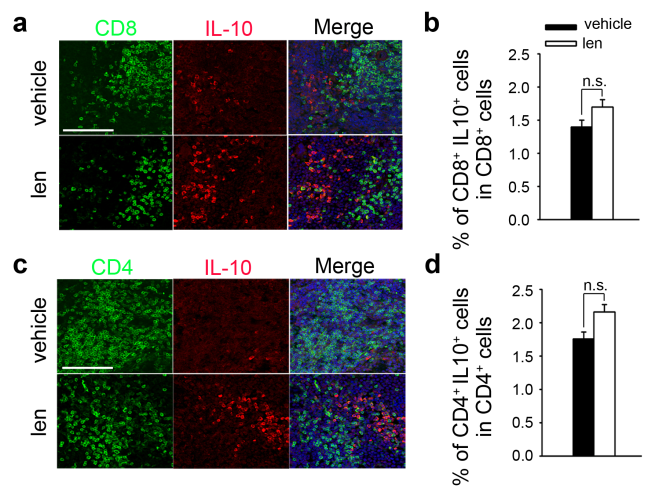


**Supplementary Figure 6** Lenalidomide-induced IL10 is not secreted by CD4^+^ or CD8^+^ T cells. (**a**) Immunostaining of mouse spinal cords from vehicle- and Lenalidomide-treated WT EAE mice at day 16 using antibodies against CD8 (green) and IL10 (red). Scale bars: 150 μm. (**b**) Quantification of the percentage of CD8^+^IL10^+^ cells among CD8^+^cells from spinal cords in (a). (**c**) Immunostaining of mouse spinal cords from vehicle- and Lenalidomide-treated WT EAE mice at day 16 using antibodies against CD4 (green) and IL10 (red). Scalebar: 150 μm. (**d**) Quantification of the percentage of CD4^+^IL10^+^ cells among CD4^+^ cells from spinal cords in (c). Data are presented as means±S.E.M.
